# Multifunctional fructans and raffinose family oligosaccharides

**DOI:** 10.3389/fpls.2013.00247

**Published:** 2013-07-09

**Authors:** Wim Van den Ende

**Affiliations:** Laboratory of Molecular Plant Biology, KU LeuvenLeuven, Belgium

**Keywords:** antioxidant, fructan, immunity, oligosaccharide, raffinose, signaling, stress, sucrose

## Abstract

Fructans and raffinose family oligosaccharides (RFOs) are the two most important classes of water-soluble carbohydrates in plants. Recent progress is summarized on their metabolism (and regulation) and on their functions in plants and in food (prebiotics, antioxidants). Interest has shifted from the classic inulin-type fructans to more complex fructans. Similarly, alternative RFOs were discovered next to the classic RFOs. Considerable progress has been made in the understanding of structure–function relationships among different kinds of plant fructan metabolizing enzymes. This helps to understand their evolution from (invertase) ancestors, and the evolution and role of so-called “defective invertases.” Both fructans and RFOs can act as reserve carbohydrates, membrane stabilizers and stress tolerance mediators. Fructan metabolism can also play a role in osmoregulation (e.g., flower opening) and source–sink relationships. Here, two novel emerging roles are highlighted. First, fructans and RFOs may contribute to overall cellular reactive oxygen species (ROS) homeostasis by specific ROS scavenging processes in the vicinity of organellar membranes (e.g., vacuole, chloroplasts). Second, it is hypothesized that small fructans and RFOs act as phloem-mobile signaling compounds under stress. It is speculated that such underlying antioxidant and oligosaccharide signaling mechanisms contribute to disease prevention in plants as well as in animals and in humans.

## INTRODUCTION

Sucrose (Suc; Glcα1,2βFru) takes a central position in plant metabolism as the first free sugar formed during photosynthesis and the major transport compound to bring carbon skeletons from source to sink tissues (Koch, 2004). Suc is the substrate for the synthesis of different types of Suc-derived oligosaccharides (Keller and Pharr, 1996). Among those, fructans and raffinose family oligosaccharides (RFOs) are the most important two classes of water-soluble carbohydrates in the plant kingdom. Fructans are fructose (Fru)-based oligo- and polysaccharides, representing the major reserve carbohydrates in about 15% of flowering plant species (Hendry, 1993). Fructans can be linear or branched and their degree of polymerization (DP) ranges from three up to a few hundred, depending on the species, developmental stage and environmental conditions (Van den Ende et al., 2002a). Fructans are classified according to differences in glycosidic linkages [β(2,1), β(2,6) or both]. The best studied fructans are the linear inulin-type fructans (occurring in Asterales such as Jerusalem artichoke and chicory) consisting of β(2,1)-linked Fru units attached to the Suc starter unit. The trisaccharide 1-kestotriose (older nomenclature 1-kestose:Glcα1,2βFru1,2βFru) is the essential building block in this case. However, Fru-only versions of these fructans, lacking a terminal glucose (Glc), and termed inulo-*n*-oses, can also be found under some conditions (Van den Ende et al., 1996). The smallest representatives of these series are inulobiose (Fru1,2βFru) and inulotriose (Fru1,2βFru1,2βFru). The building block for the linear levan-type fructans (also termed phleins in plants) is 6-kestotriose (older nomenclature 6-kestose: Glcα1,2βFru6,2βFru) which is further elongated to polymers with β(2,6)-linkages. They typically occur in forage grasses such as *Dactylis glomerata*, *Phleum pratense*, and *Poa secunda* (Chatterton et al., 1993; Chatterton and Harrison, 1997; Tamura et al., 2009). Graminan-type fructans contain both β(2,1) and β(2,6) linkages. They occur in cereals such as wheat and barley (Van den Ende et al., 2003). Even more complex fructans, based on the 6G-kestotriose backbone (older nomenclature neokestose: Fru2,6Glcα1,2βFru), with further Fru elongations on both sides, occur for instance in oat, *Asparagus*, *Agave*, and in *Lolium* sp. (Pavis et al., 2001). These are termed neo-inulin [predominant β(2,1) linkages] and neo-levan-type [predominant β (2,6) linkages] fructans, respectively. The longstanding view that graminan- and levan-type fructans only occur in monocots has been overruled, since both types have been recently found in *Pachysandra terminalis*, an evergreen, frost-hardy basal eudicot species (Van den Ende et al., 2011a). Although some suggestions were made to link fructan structure to functionality in stress tolerance responses (Valluru and Van den Ende, 2008; Lasseur et al., 2011; Van den Ende et al., 2011a), these relationships are unclear and require further experimental verification. Fructans are believed to accumulate in vacuoles (Wiemken et al., 1986) but it was proposed that, under stress, tonoplast-derived vesicles may transport fructans from the vacuole to the apoplast (Livingston and Henson, 1998; Valluru et al., 2008).

The “classic” RFOs are soluble, non-reducing α(1,6) galactosyl (Gal) extensions of Suc. The trisaccharide raffinose (Raf; Galα1,6Glcα1,2βFru) is the smallest RFO and ubiquitous in the plant kingdom (Keller and Pharr, 1996). Further elongation with Gal residues leads to the DP4 stachyose (Sta; Galα1,6Galα1,6Glcα1,2βFru), verbascose (DP5), ajugose (DP6), etc. Classic RFOs with a DP up to 15 have been found after cold treatment in *Ajuga reptans* L. (Bachmann et al., 1994), a typical RFO accumulator belonging to the *Lamiaceae*. While Raf and Sta occur in all plant parts in genuine RFO accumulators, the higher homologous are usually restricted to the storage organs. Often Sta is the quantitatively dominating carbohydrate in such storage organs (Kandler and Hopf, 1984). Raf and Sta are also important transport compounds in the orders *Lamiales*, *Cucurbitales*, *Cornales*, and in one family of the *Celastrales* (Zimmermann and Ziegler, 1975; Haritatos et al*.*, 1996; Hoffmann-Thoma et al., 1996; Turgeon et al*.,* 2001). Recently, research has been devoted to so-called “alternative” RFOs in plants. These novel plant Gal oligosaccharides did not derive much attention in the past. Among these, the Sta derivative manninotriose (Galα1,6Galα1,6Glc) was found to be the predominant carbohydrate in cold-induced early spring red deadnettle (dos Santos et al., 2013), a unique feature since this compound was never observed before in any RFO accumulator (dos Santos et al., 2013). Intriguingly, Sta does not occur within the *Caryophyllaceae.* Instead, Raf is elongated to the DP4 lychnose (Galα1,6Glcα1,2βFru1,1Gal) and the DP5 stellariose ([6Galα1,6&Galα1,4]Glcα1,2βFru1,1Gal) in cold-treated *Stellaria media* (Vanhaecke et al., 2006, 2008, 2010).

## METABOLISM AND ITS REGULATION

Sucrose is not only needed as a substrate for fructan biosynthetic enzymes (termed fructosyltransferases: FTs), organ-specific Suc thresholds trigger the expression of genes encoding FTs (Lu et al., 2002; Maleux and Van den Ende, 2007) and RFO biosynthesis genes (Nägele and Heyer, 2013). Similar to the induction of anthocyanins in *Arabidopsis* (a non-fructan accumulator), it is well-known that fructan synthesis is controlled by a Suc-specific pathway (Bolouri Moghaddam and Van den Ende, 2013a and references therein), which means that the same effects cannot be obtained by using a mixture of Glc and Fru. Calcium, protein kinases and phosphatases are also involved in this inductive process (Martínez-Noël et al., 2009, 2010). Recently, the transcription factor TaMYB13 was found to be an important player in the process leading to FT induction and fructan synthesis in wheat (Xue et al., 2011) but further research into this pathway is needed to fully understand where this transcription factor is situated in the pathway. Even less is known about the pathway leading to RFO synthesis. However, it seems that heat shock transcription factors (HSFs), C-repeat binding factor/drought response element binding factor 1 (CBF/DREB1) type transcription factors and WRKY type of transcription factors (Panikulangara et al., 2004; Ogawa et al., 2007; Wang et al., 2009). Recently, it was reported that target of rapamycin kinase complexes stimulate the pathway leading to RFO synthesis in *Arabidopsis* (Dobrenel et al., 2013 and references therein).

Inulin-type fructans are biosynthesized from Suc by two FTs. First, 1-kestotriose is produced by the activity of a sucrose:sucrose 1-fructosyl transferase (1-SST) which transfers a fructosyl residue from a donor to an acceptor Suc. Then, a fructan:fructan 1-fructosyl transferase (1-FFT) polymerizes 1-kestotriose into higher DP inulin-type fructans (Edelman and Jefford, 1968; Van Laere and Van den Ende, 2002). Sucrose:fructan 6-fructosyl transferases (6-SFTs) are able to introduce branching. They preferentially transfer a fructosyl group from Suc as a donor substrate to 1-kestotriose as acceptor substrate, producing 1&6-kestotetraose (also termed bifurcose), the smallest graminan-type of fructan with mixed-type of linkages. Bifurcose can be further elongated by 6-SFT and 1-FFT, leading to branched, higher DP graminan-type of fructans (Yoshida et al., 2007). However, some of these 6-SFT enzymes might use Suc and/or 6-kestotriose as preferential acceptors, producing levan-type fructans (Tamura et al., 2009). Such 6-SST/6-SFT is also involved in fructan synthesis in *Pachysandra terminalis*, although this particular enzyme also shows extensive hydrolytic activities as well (Van den Ende et al., 2011a; Lammens et al., 2012), and it can be considered as a “premature” FT [preliminary fructosyl transferase (pFT); see also below]. Finally, the enzyme fructan:fructan 6G-fructosyl transferase (6G-FFT) synthesizes 6G-kestotriose (neokestose) from 1-kestotriose as donor substrate and Suc as acceptor substrate. Further elongation by 1-FFT and 6-SFT leads to the formation of inulin- and levan neoseries, respectively (Vijn and Smeekens, 1999). Plants use an array of different fructan exohydrolases (FEHs) to degrade their fructans (Van den Ende et al., 2004; Yoshida et al., 2007; Zhang et al., 2008), including 1-FEHs [preferentially attacking β(2,1) Fru linkages], 6-FEHs [preferentially attacking β (2,6) Fru linkages] and 6&1-FEHs (attacking both types of linkages). These enzymes remove, one by one, terminal Fru units from fructan chains. In contrast to invertases, FEHs cannot use Suc as a substrate. Instead, many FEHs are directly inhibited by Suc at the enzyme level (Verhaest et al., 2007), which represents one of the most important ways of regulation, next to the control of FEH gene expression at the transcriptional level (Van den Ende et al., 2002a). Remarkably, some of the apoplastic localized FEHs show an extreme specificity for single fructan kestotrioses, and these are termed kestotriose exohydrolases (Van den Ende et al., 2005), indicating that these forms might play a role in fructan signaling events (Van den Ende et al., 2004). It is known since long that FEHs also occur in non-fructan accumulators. However, they are probably better considered as “defective invertases” with possible (artificial) FEH side activities. The role of these proteins remained enigmatic for a very long period. However, a recent breakthrough paper (Le Roy et al., 2013) shows that Nin88, an apoplastic defective invertase from tobacco lacking FEH side activities, acts as indirect activator of active cell wall invertases (CWIs) which are crucial players in overall plant development, especially seed and fruit setting (Ruan et al., 2012). Although the exact underlying regulatory mechanisms require further research, data indicate that Nin88 interacts with cell walls in such a way that active CWIs bind to the cell wall in a more productive way (Le Roy et al., 2013). This fits nicely within the emerging concept that dead enzymes are very common in all kingdoms of life and that many of them fulfil crucial biological roles, as reviewed in a recent Science paper (Leslie, 2013).

The first committed step in RFO biosynthesis is the production of galactinol (Gol) from *myo*-inositol and UDP-Gal, a reaction catalyzed by galactinol synthase (GolS; Keller and Pharr, 1996). Next, Gol is used as a donor to deliver Gal to Suc, creating Raf. This is catalyzed by raffinose synthase (RafS). Stachyose synthase uses Gol as donor and Raf as acceptor to synthesize Sta (Keller and Pharr, 1996). GolS, RafS, and StaS are believed to localize in the cytosol, although the RFOs they produce might also enter the vacuole and the chloroplasts (Nägele and Heyer, 2013). In some species, higher DP RFOs are produced by the action of galactan:galactan galactosyl transferases (GGTs; Bachmann et al., 1994), using RFOs as donor and acceptor substrates. Although the exact origin of manninotriose type of RFO in red deadnettle is not known, it was suggested that this compound results from invertase (β-fructosidase) activity on Sta (dos Santos et al., 2013). Lychnose synthase and stellariose synthase are the enzymes involved in the biosynthesis of lychnose and stellariose (Vanhaecke et al., 2010). RFO catabolism involves the activity of acid and alkaline α-galactosidases which sequentially remove the terminal Gal residues (Keller and Pharr, 1996), while β-fructosidases may produce melibiose (Galα1,6Glc) from Raf and manninotriose from Sta (dos Santos et al., 2013). The so-called seed imbibition proteins resemble the enzymes involved in RFO catabolism, but only a few forms have been functionally characterized (Peters et al., 2010). Similar to defective invertases, it can be speculated that some of these forms may represent catalytically inactive forms, acting as regulatory proteins. Some forms may be involved in the degradation of RFOs acting as cellular signals (see below).

## ENZYMES: STRUCTURE–FUNCTION RELATIONSHIPS

The overall classification into families of carbohydrate active enzymes^1^ is based on amino acid sequence similarities (Cantarel et al., 2009). This classification (i) reflects the structural features of these enzymes better than their sole substrate specificity, (ii) helps to reveal the evolutionary relationship between these enzymes, and (iii) provides a convenient framework to understand mechanistic properties (Henrissat and Romeu, 1995).

Plant acid invertases (β-fructosidases), including vacuolar invertases (VIs) and CWIs, split Suc into Fru and Glc by hydrolysis of the glycosidic bond. FEHs hydrolyze a terminal Fru from a fructan chain, while FTs elongate a Suc or fructan molecule with an extra Fru moiety. Taken together, all these enzymes transfer a Fru unit either to water (hydrolysis), to Suc or fructan (Van den Ende et al., 2009). They only differ in their specificity for donor and acceptor substrates. Accordingly, the 3D structure determinations of a FEH from chicory (Verhaest et al., 2005), a CWI from *Arabidopsis* (Lammens et al., 2008) and a pFT from *Pachysandra terminalis* (Lammens et al., 2012) showed that all these enzymes (or proteins: defective invertases) have a common fold. Hence, they are grouped together with microbial β-fructosidases (degrading both Suc and fructans) in the family 32 of glycoside hydrolases (GH32). Family GH32 is combined with family GH68 in the clan GH-J. GH68 harbors bacterial invertases, levansucrases and inulosucrases. All these proteins consist of an N-terminal five-bladed β-propeller domain (GH32 and GH68) followed by a C-terminal domain formed by two β-sheets (only in GH32). The active site is present within the β-propeller domain and characterized by the presence of three highly conserved acidic groups (present in the WMN**D**PNG, R**D**P, and **E**C motifs). The Asp from the first motif is acting as nucleophile, the Asp from the second motif is believed to be a transition state stabilizer and the Glu residue from the **E**C motif acts as acid/base catalyst playing a crucial role in the catalytic mechanism (Van den Ende et al., 2009). Some sugars can bind as substrates or as inhibitors in the active site of plant GH32 members (Verhaest et al., 2007) and this depends on subtle amino acid variations in the active site area. Recent pKa calculations suggest that most GH-J members show an acid–base catalyst that is not sufficiently protonated before ligand entrance, while the acid–base can be fully protonated when a substrate, but not an inhibitor, enters the catalytic pocket (Yuan et al., 2012). Moreover, the conserved arginine in the RDP motif, rather than a previously proposed Tyr in the FYASK motif, is proposed to play a key role to increase the pKa of the acid–base catalyst (Yuan et al., 2012).

Intriguingly, defective invertases are never affected in their catalytic triad, but rather in a neighboring “Asp/Lys” or “Asp/Arg couple” (present in a flexible loop in the proximity of the acid/base catalyst) and in some Trp residues (Le Roy et al., 2007, 2013). These residues are essential to stabilize the Glc part of Suc in the active site of GH32 Suc splitting enzymes (CWINV, VI, 1-SST, 6-SFT; Van den Ende et al., 2009), and they are absent in enzymes that use fructans as donor substrates (FEH, 1-FFT, 6G-FFT). This was confirmed by site directed mutagenesis experiments on invertase, defective invertase, FEH and 6G-FFT (Le Roy et al., 2007, 2008, 2013; Lasseur et al., 2009). However, the presence of an Asp/Lys or Asp/Arg is not sufficient; this couple needs to be in the right 3D configuration as well (Schroeven et al., 2009). The recent 3D structure of *Pachysandra terminalis* with its acceptor substrate 6-kestotriose strongly suggested that the couple (Asp/Gln in this case) plays a prominent role in acceptor substrate specificity as well (Lammens et al., 2012).

All RFO metabolizing enzymes discussed in the previous section, with the exception of GolS, belong to GH27 and GH36 in clan D. The acid α-galactosidases and GGTs are grouped into GH27, where some 3D structures have been determined, including the acid α-galactosidase from rice (Fujimoto et al., 2003). Their active sites are well-conserved and formed by residues in the loops at the ends of the β-strands in a (β/α)_8_ barrel. Two Asp residues are required for catalysis, which are positioned on opposite sides of the labile glycosidic bond (Fujimoto et al., 2003). RafS, StaS, and alkaline α-galactosidases belong to the related GH36, but no structural information is yet available on plant members within this family (Vanhaecke, 2010), although a few microbial structures became available (Fredslund et al., 2011; Merceron et al., 2012). To our knowledge, no in depth structure–function research has been performed toward donor and acceptor substrate specificities within plant members of GH27 and GH36. Clearly, such studies would be very informative as well. Such insights greatly contribute to rational enzyme design contributing to the production of tailor-made fructans and RFOs.

## EVOLUTION

**FIGURE 1:**
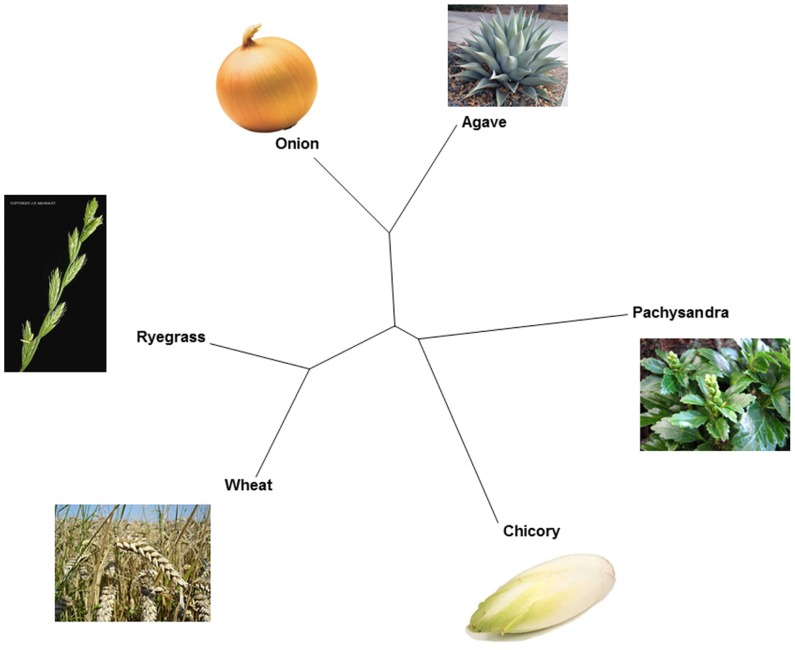
**Fructan diversity and polyphyletic origin of fructan biosynthesis in higher plants.** An unrooted phylogenetic tree of fructan initiator enzymes (1-SSTs and a pFT) derived from six well-known fructan accumulators (chicory, wheat, ryegrass, onion, Agave, and Pachysandra) is drawn to illustrate the polyphyletic origin of fructan biosynthesis in higher plants.

**FIGURE 2:**
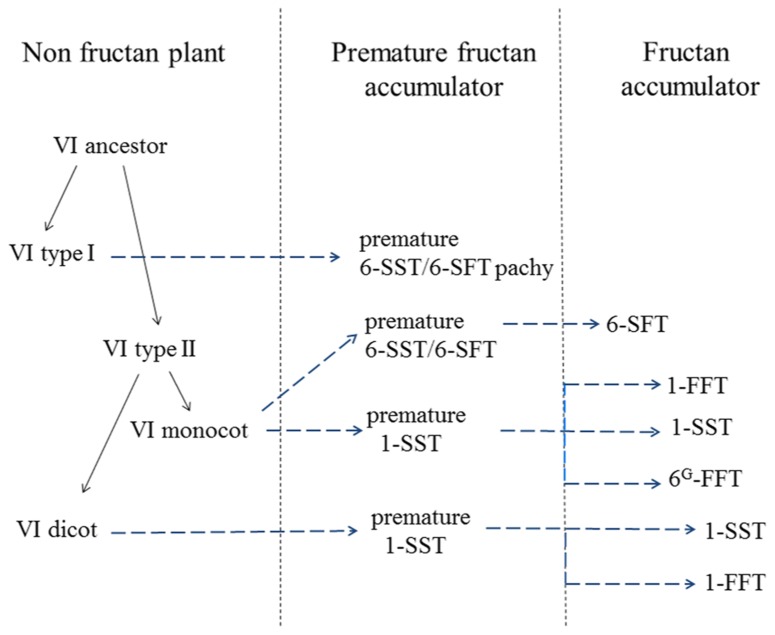
**Model depicting the putative evolution of different higher plant fructosyl transferases.** Likely, an ancestral VI duplicated in two VI types (I and II) in a non-fructan-accumulating plant, probably before the separation of monocots and dicots. Within the basal eudicots, a type I VI was recruited to create a preliminary FT (a 6-SST/6-SFT) in *Pachysandra terminalis*, a recent evolutionary event. During older evolutionary events, certain monocot and dicot type II VIs evolved into preliminary 1-SSTs and 6-SST/6-SFTs that later specialized into genuine, more specific 1-SSTs and 6-SFTs. Likely, 1-FFTs (dicots) and 6G-FFTs (monocots) evolved later, based on (preliminary) 1-SST precursors. See text for more details.

Within GH32, it became clear that plant FTs evolved from VIs (Wei and Chatterton, 2001; Altenbach et al., 2009), contributing to the observed diversity in fructan accumulators in the plant kingdom (**Figure 1**). Two types of VIs (I and II, Van den Ende et al., 2002b) can be discerned in plants and for a long time it was assumed that all plant FTs evolved from (different forms of) type II VIs. This occurred at least three times: (i) in the Asterales (inulin-type of fructans; e.g., chicory, (ii) in the Poales with further distinction between cool-season grasses (mainly levan and neokestose-derived fructans, e.g., ryegrass) and cereals (predominantly graminan-type fructans, e.g., wheat and barley) in the Poaceae and (iii) in the Asparagales further splitting into the Allioideae (e.g., onion) and Agavoideae (e.g., Agave) subfamilies that also mainly accumulate neokestose-based fructans (**Figure 1**). However, this view was changed by the unexpected discovery of both levan- and graminan-type fructans in the basal eudicot *Pachysandra terminalis* species, containing a pFT that, surprisingly, evolved from a type I VI (**Figure 2**) and not from a type II VI as observed for all other FTs (**Figure 2**). This further confirmed the polyphyletic origin of fructan biosynthesis (Altenbach et al., 2009; Van den Ende et al., 2011a) and suggests that the capacity for fructan biosynthesis arose at least four times during the plant diversification process (**Figure 1**). Such polyphyletic origin did not likely occur within GH27 and GH36, although more sequences should be generated to reach this conclusion (Vanhaecke, 2010). By combining alignments, 3D structure information and phylogenetic analyses (Schroeven et al., 2008; Altenbach et al., 2009; Lasseur et al., 2011; Lammens et al., 2012), the current view within GH32 is that an ancestral VI duplicated in two VI types (I and II, **Figure 2**) before the separation of monocots and dicots (Wei and Chatterton, 2001). Most probably, monocot and dicot type II VIs were than recruited to create preliminary 1-SSTs and 6-SFTs that later specialized into genuine 1-SSTs and 6-SFTs (**Figure 2**). Mutations in the “WMNDPNG” and “W(A/G)W” motifs are believed to play a key role in such processes (Schroeven et al., 2008, 2009; Altenbach et al., 2009). In evolutionary terms, it seems reasonable to assume that, in monocots as well as in dicots, 1-FFTs and 6G-FFTs evolved later, likely from (premature) 1-SST precursors (**Figure 2**). For instance, in wheat the identity between Ta1-SST and Ta1-FFT is much higher (84%) than between Ta1-SST and TaVI (67%) and between Ta1-FFT and TaVI (66%), strongly suggesting that Ta1-FFT evolved from Ta1-SST (**Figure 2**; Schroeven et al., 2009). A similar reasoning led to the hypothesis that the *Lolium perenne* Lp6^G^-FFT evolved from a (premature) Lp1-SST (**Figure 2**; Lasseur et al., 2009). In the same way, it can be speculated that the chicory Ci1-FFT evolved from a (premature) Ci1-SST (**Figure 2**; Schroeven et al., 2009). Within the basal eudicots, a type I VI developed into a pFT in *Pachysandra terminalis* (**Figure 2**) and this is considered as a rather “recent” evolutionary event (Van den Ende et al., 2011a). On the contrary, defective invertases and FEHs evolved from CWIs within GH32 (Le Roy et al., 2007, 2013). It can be speculated that the loss or alteration of the above-mentioned “couple” is an early evolutionary event that led to the formation of defective invertases with cell wall localization and a high iso-electric point (pI) for interaction with the cell wall. To further develop genuine FEHs in fructan plants, it can be further hypothesized that precursor defective invertases retrieved (i) a vacuolar targeting signal for sorting to the central vacuole, (ii) a low pI typical for vacuolar proteins, (iii) amino acid alterations that helped stabilization of higher DP fructans as donor substrates (Le Roy et al., 2008).

## CLASSIC FUNCTIONS OF FRUCTANS AND RFOs

The most widely accepted function of fructans is their role as a storage carbohydrate. Dicots typically store inulin-type fructans in underground reserve organs (roots, tubers) (Van Laere and Van den Ende, 2002) while monocots typically store fructans on a shorter term basis in above ground parts of the plant (Pollock and Cairns, 1991; Slewinski, 2012). To the best of our knowledge, fructans are the only neutral type of polysaccharides that accumulate in plant vacuoles. Fructans can accumulate to 20% on fresh weight basis and even up to 70% on a dry weight basis in some organs (Wiemken et al., 1995). Trying to solubilize such levels *in vitro* invariably leads to fructan precipitation, suggesting that fructans *in vivo* should be organized in a special way to keep them in a (semi)-soluble condition in the vacuole (Van den Ende, 1996). It is clear that starch is the most widespread reserve carbohydrate in the plant kingdom. On the one hand, insoluble starch granules represent a very elegant way of storing huge amounts of carbon in a very small volume. On the other hand, excessive amounts of water-insoluble starch would be physically destructive to the chloroplast, its site of synthesis and storage in leaves. Therefore, fructans may have some advantages as compared to starch. One of the arguments in favor of using fructans could be the fact that starch biosynthesis dramatically decreases when the temperature drops below 10°C, whereas fructan biosynthesis is much less sensitive to low temperatures (Pollock, 1986). Another difference between starch and fructans might include the speed of its breakdown and carbon remobilization. While a large array of different enzymes (dikinases, phosphatases, starch hydrolases) are necessary to release small sugars from a starch granule (Stitt and Zeeman, 2012), water-soluble fructans are expected to be degraded much quicker by the action of FEHs as a single enzyme type. In grasses fructans are mainly stored in the leaf bases and used for regrowth after defoliation (Morvan-Bertrand et al., 2001). In cereals, fructans temporarily accumulate in stems and early in seed development (Van den Ende et al., 2003; Van den Ende et al., 2011b; Joudi et al., 2012) as well as in reproductive organs (Ji et al., 2010). Contrary to the situation in dicots, where growth and fructan accumulation are usually separated in time, monocots are able to combine these processes. It could be argued that the activity of Suc splitting enzymes 1-SST and 6-SFT contribute to control and maintain sink strength and carbohydrate supplies (Ji et al., 2010), but then the obvious question can be raised why this is not simply accomplished by increasing the activity of invertases? This indicates that the accumulation of fructans as such should somehow be beneficial (see also below), especially under stress.

Fructans can also play a role during flower opening. Fructan contents are high in closed petals of *Campanula rapunculoides* and *Hemerocallis* while no fructan is present anymore in petals of opened flowers (Bieleski, 1993; Vergauwen et al., 2000). FEHs quickly release massive amounts of Fru, lowering the osmotic potential and contributing to water inflow and flower opening. Fructans appear to have additional functions in drought, salt, and freezing tolerance of plants (Valluru and Van den Ende, 2008; Livingston et al., 2009). This is further supported by the fact that fructan-accumulating plants are especially abundant in temperate and arid climate zones with seasonal frost or drought periods, and are almost absent in tropical regions (Hendry, 1993). In fructan species, fructan accumulation can be induced under drought (De Roover et al., 2000) and cold (Livingston and Henson, 1998; Yoshida et al., 2007). More direct evidence comes from the observation that fructan-accumulating transgenic plants show enhanced stress tolerance (Pilon-Smits et al., 1995, 1999; Konstantinova et al., 2002; Li et al., 2007; Kawakami et al., 2008; Bie et al., 2012). Transgenic perennial ryegrass expressing wheat 1-SST or 6-SFT genes accumulate more fructans and acquired higher tolerance for freezing at the cellular level (Hisano et al., 2004). Therefore, it would be interesting to introduce FT genes in a number of food and biomass crops, to make them more tolerant to abiotic stresses.

Next to fructans, RFOs are also used as “storage carbohydrates,” arbitrarily defined as those which occur at more than 1% of the dry weight of a given tissue. So, despite the fact that most plants synthesize RFOs (at least Raf) to some extent at some stage of their development, only some plants accumulate large amounts of them (Kandler and Hopf, 1984; Keller and Pharr, 1996). These RFO accumulators store RFOs in concentrations up to 25–80% of their dry weight in specialized storage organs such as tubers (e.g., *Stachys sieboldii*), seeds (e.g., soybean, lentil, chickpea), or in photosynthesizing leaves (e.g., *Ajuga reptans*; Bachmann et al., 1994; Tahir et al., 2012). Similar to fructans, and in contrast to starch, RFOs are osmotically flexible as their DP may easily change and so the osmotic pressure. Species that use Suc as reserve carbohydrate (sugar beet, sugar cane) can only double the osmotic pressure upon hydrolysis (Gilbert et al., 1997). Finally, RFOs are phloem-mobile, and are readily available for carbon translocation when required. This feature is less clear for fructans, since phloem mobility has only been documented in a single fructan accumulator (see below). Typically, strong RFO and fructan accumulation do not occur together in a single plant species, suggesting that RFOs and fructans might fulfill similar (or partially overlapping) physiological functions. To better understand subtle differences in their physiological functions, it would be interesting to seek for plants that are capable to store high levels of both RFOs and fructans. Similar to the introduction of FTs in non-fructan accumulators, the overexpression of GolS in *Arabidopsis thaliana* resulted in plants with increased Raf levels and increased stress tolerance (Taji et al., 2002; Nishizawa et al., 2008). This suggests that the presence of increased levels of fructans or RFOs (in plants that normally contain very low or undetectable levels of such components) helps plants to survive adverse climatic conditions.

## MEMBRANE STABILIZATION AND ANTIOXIDANT PROPERTIES

What could be the underlying mechanisms to explain such increased stress tolerances? Since membranes (and critical membrane proteins) are one of the primary targets of freezing and desiccation injury in cells (Oliver et al., 2000), membrane protective effects have been dedicated to fructans as well as to RFOs. *In vitro* experiments provided evidence for this ability, demonstrating that both fructans and RFOs contribute to enhanced membrane stability during freezing and cellular dehydration by deep insertion between the headgroups of lipids, both in mono- and bilayers (Demel et al., 1998; Vereyken et al., 2001; Hincha et al., 2002, 2003; Valluru and Van den Ende, 2008; Valluru et al., 2008). As such, they are also well-positioned to scavenge hydroxyl radicals (^·^OH) which might originate from tonoplast-associated Class III peroxidase activities (Passardi et al., 2004; Van den Ende and Valluru, 2009). Among the biologically relevant reactive oxygen species (ROS: H_2_O_2_ $O_{2}^{\cdot -}$ and ^·^OH), hydroxyl radicals are the most reactive and dangerous species (Keunen et al., 2013). The ^·^OH is known to react with almost all biomolecules at rates as those occurring in diffusion-controlled reactions (Hernandez-Marin and Martínez, 2012). As a consequence there are no enzymatic systems known to neutralize them in any living beings (Gechev et al., 2006). The *in vitro* ^·^OH scavenging activity of Raf and fructans has recently been confirmed (Stoyanova et al., 2011, Peshev et al., 2013) and compared to an array of phenolic compounds, well-known superior antioxidants (Peshev et al., 2013). Based on these findings, a hypothetical model has been proposed explaining how vacuolar fructans and phenolic compounds may act in a synergistic way to contribute to vacuolar antioxidant mechanisms *in vivo,* and to overall cellular homeostasis (Peshev et al., 2013). While fructans are obvious candidates for tonoplast stabilization and protection, RFOs (Raf in cold-induced *Arabidopsis* leaves) that are synthesized in the cytosol are candidates to protect the plasma membrane. However, this seems not to be the target membrane in *Arabidopsis* (Nägele and Heyer, 2013). Instead, it was demonstrated that Raf specifically acts to protect the photosystems located in the thylakoid membranes of plastids from damage during freeze thaw cycles (Knaupp et al., 2011). It was recently demonstrated that Raf can be imported in chloroplasts (Schneider and Keller, 2009) and therefore it could function as a cryoprotectant. As explained above for fructans or other osmolytes, it can be speculated that the ^·^OH scavenging capacity of Raf counteracts membrane and protein damage, contributing to thylakoid membrane stability and chloroplast integrity under stress (Doltchinkova et al., 2013). Likewise, targeting the synthesis of mannitol, another well-known ^·^OH scavenger (Stoyanova et al., 2011), to chloroplasts resulted in increased resistance to oxidative stress (Shen et al., 1997a, b), similar to what is observed in GolS overexpressors with their increased Raf levels (Nishizawa et al., 2008).

## SIGNALING?

Nowadays, Glc, Fru, and Suc-specific signaling pathways have been elucidated in plants (Rolland et al., 2006; Cho and Yoo, 2011; Li et al., 2011), already suggesting that a signaling role for other types of small endogenous sugars should not be simply neglected. It seems that (a) Suc-specific signaling pathway(s) contributes to plant defense responses (Bolouri Moghaddam and Van den Ende, 2013a, b). Increased Suc levels typically lead to increased levels of fructans, RFOs and/or anthocyanins (Teng et al., 2005; Martínez-Noël et al., 2009, 2010; Nägele and Heyer, 2013), perhaps controlled by (a single) Suc-specific signaling pathway(s) (Bolouri Moghaddam and Van den Ende, 2013a).

Gol and Raf are now recognized as signaling molecules during biotic stress responses (Kim et al., 2008) and a similar role during abiotic stress responses has been suggested for RFOs (Valluru and Van den Ende, 2011; Eyles et al., 2013) and for fructans (Van den Ende et al., 2004). This led to the hypothesis that both RFOs and small fructans might act as endogenous, phloem-mobile stress signals. Indeed, small fructans have been detected in the phloem sap of Agave (Wang and Nobel, 1998) and it was reported that the fructan 6-kestotriose is phloem-mobile when it is produced by yeast invertase expressed in companion cells (Zuther et al., 2004). According to this view, the small fructans 1-kestotriose^2^ and its derivative inulobiose^3^ have been recently detected at very low levels in *Arabidopsis*, widely known as a strict non-fructan accumulator. What could be the origin of these small fructans in healthy *Arabidopsis* tissues? The most straightforward explanation is that these fructans are produced by the activities of VIs (AtVI1 and AtVI2), since *Arabidopsis* is lacking genuine FTs. *Arabidopsis* VIs were isolated before and found to contain considerable FT activities when tested at high Suc levels (De Coninck et al., 2005). Thus, next to Suc signaling, RFO and fructan signaling concepts should not be neglected and become the subject of intensive investigations. Possibly, such signaling events form the basis of the so-called “sugar-based resistance” or “sweet immunity” concept (Gomez-Ariza et al., 2007; Bolouri Moghaddam and Van den Ende, 2012, 2013b) in plants, but perhaps also in animals (see below).

## UNIVERSAL IMMUNOSTIMULATORS?

Interest in fructans and RFOs increased during the last decade due to their health-promoting effects, selectively stimulating beneficial bacteria, acting as prebiotics (Shoaf et al., 2006; Kolida et al., 2007). These effects may be indirectly mediated through their fermentation products, but direct effects should not be neglected (Van den Ende et al., 2011b; Di Bartolomeo et al., 2013). Inulin-type fructans and fructo-oligosaccharides are the most studied and widely applied prebiotics isolated from chicory roots, and added to a variety of food products (Roberfroid et al., 2010). However, attention is shifting to longer DP and branched-type fructans (e.g., wheat graminans and Agave fructans) and RFOs as superior prebiotics (Urias-Silvas et al., 2008; Vanhaecke, 2010; Casiraghi et al., 2011; Jenkins et al., 2011), since these may provide a better “protection” (lowered colon cancer risk) over the whole length of the colon (Di Bartolomeo et al., 2013). Besides their prebiotic characteristics, fructans and RFOs are also emerging as important immunostimulators in animals and humans (Hoentjen et al., 2005; Seifert and Watzl, 2007; Vos, 2008; Delgado et al., 2012; Lee et al., 2012), as they likely do in plants (Bolouri Moghaddam and Van den Ende, 2012, 2013b). Taken all together, it can be speculated that these oligosaccharides may be involved in universal antioxidant and immunostimulatory mechanisms in plants, animals, humans, and perhaps in all eukaryotic organisms, but this requires further investigations. Needless to say, understanding the underlying mechanisms could greatly contribute to disease prevention strategies, both in plants and in mammals (Van den Ende et al., 2011b; Bolouri Moghaddam and Van den Ende, 2012, 2013b; Di Bartolomeo et al., 2013).

## Conflict of Interest Statement

The author declare that the research was conducted in the absence of any commercial or financial relationships that could be construed as a potential conflict of interest.

## Footnotes

1 http://www.cazy.org/

2 http://gmd.mpimp-golm.mpg.de/Analytes/5578B0AB-DAA3-4DC7-80A9-321065427188.aspx

3 http://gmd.mpimp-golm.mpg.de/Analytes/5578B0AB-DAA3-4DC7-80A9-321065427188.aspx

## ABBREVIATIONS

1-FFT: fructan:fructan 1-fructosyl transferase
1-SST: sucrose:sucrose 1-fructosyl transferase
6G-FFT: fructan:fructan 6G-fructosyl transferase
6-SFT: sucrose:fructan 6-fructosyl transferase
CWIs: cell wall invertases
DP: degree of polymerization
FEH: sucrose:fructan 6-fructosyl transferase
Fru: fructose
FTs: fructosyltransferases
Gal: galactose
GGTs: galactan:galactan galactosyl transferases
GHXX: family XX of glycoside hydrolases
Glc: glucose
Gol: galactinol
GolS: galactinol synthase
pFT: preliminary fructosyl transferase
pI: iso-electric point
Raf: raffinose
RafS: raffinose synthase
RFOs: Raffinose Family Oligosaccharides
ROS: reactive oxygen species
Sta: stachyose
StaS: stachyose synthase
Suc: sucrose
VIs: vacuolar invertases
